# Supplementary material for: Changes in the tumor microenvironment and outcome for TME-targeting therapy in glioblastoma: A pilot study
Source: PLoS One. 2021 Feb 5;16(2):e0246646. doi: 10.1371/journal.pone.0246646 (PMC7864405; doi:10.1371/journal.pone.0246646)
Supplement: S1 File — (DOCX) [file pone.0246646.s001.docx]

**
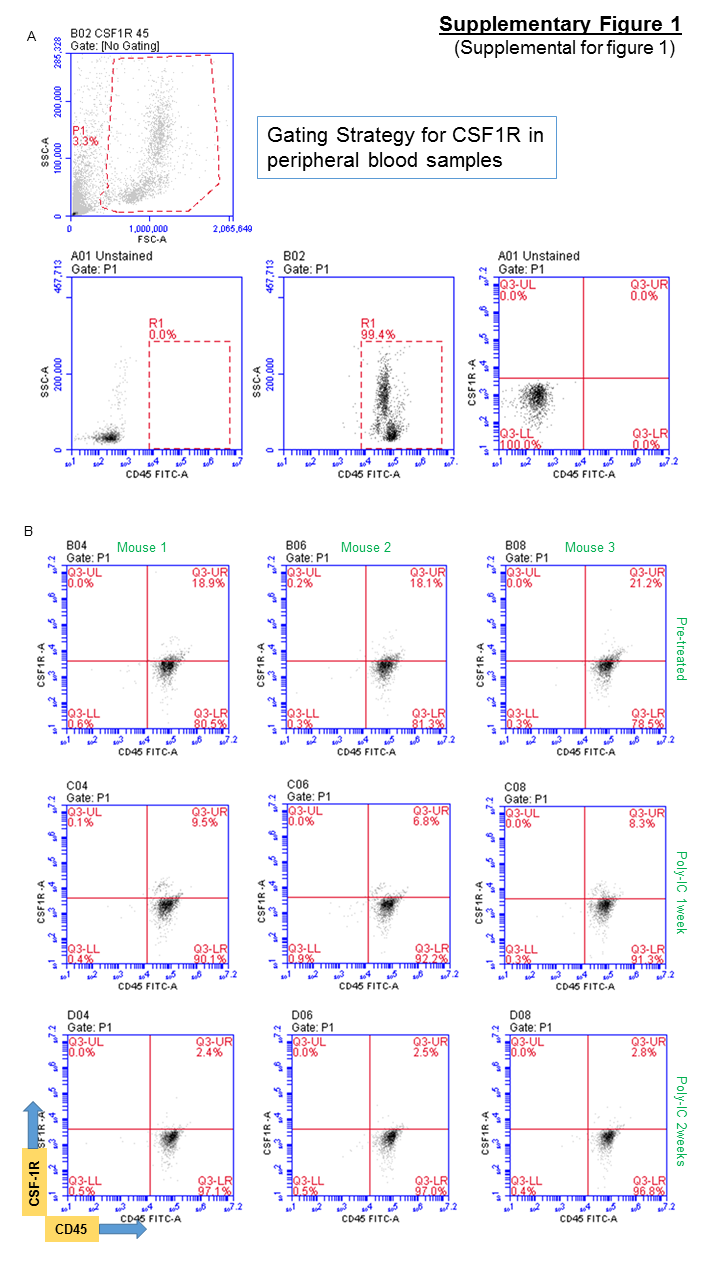
**

**S1 Fig**: (Corresponds Fig 1) **(A)** Gating Strategy for CSF1R in peripheral blood samples. **(B)** Representative dot plots of flow-cytometric analysis of peripheral blood cells from conditional knock out mice showed a significant dose-dependent decrease in CD45+CSF1R+ cells following two weeks of treatments with poly-IC.

**
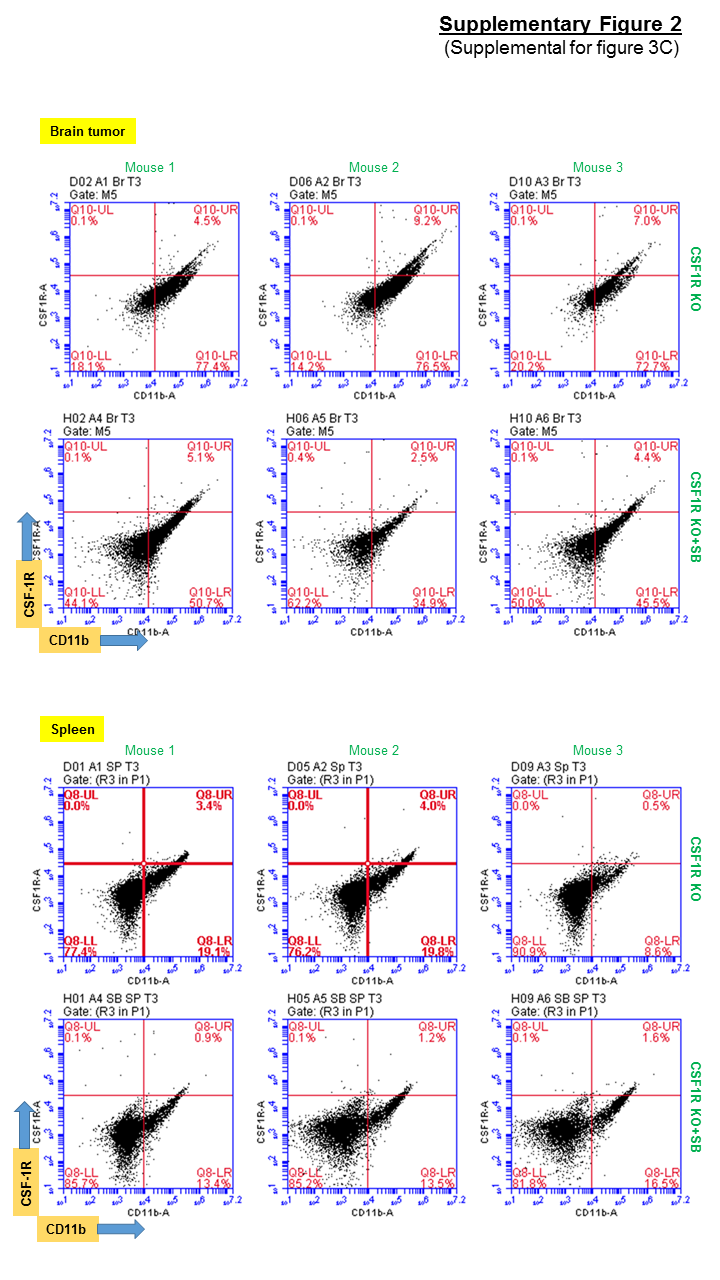
**

**S2 Fig**: (Corresponds Fig 3) Representative dot plots of flow-cytometric analysis showing a significant decrease in CSF1R+ cells in brain tumor (upper panel) and spleen (lower panel) of the knockout mice compared to the wild type mice treated with vehicle.

**
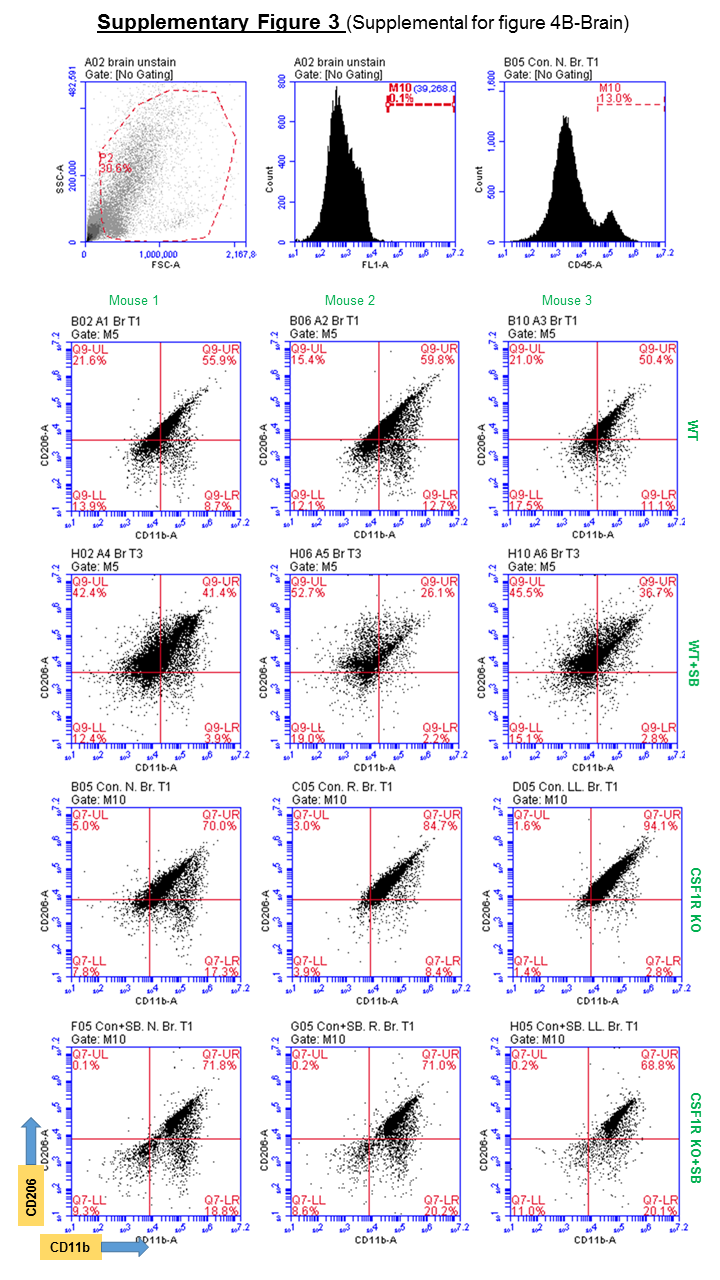
**

**S3 Fig**: (Corresponds Fig 4) Representative dot plots of flow-cytometric analysis showing a significant decrease of TAMs (CD45+CD11b+CD206+) cells in the brain following the treatment with SB225002.

**
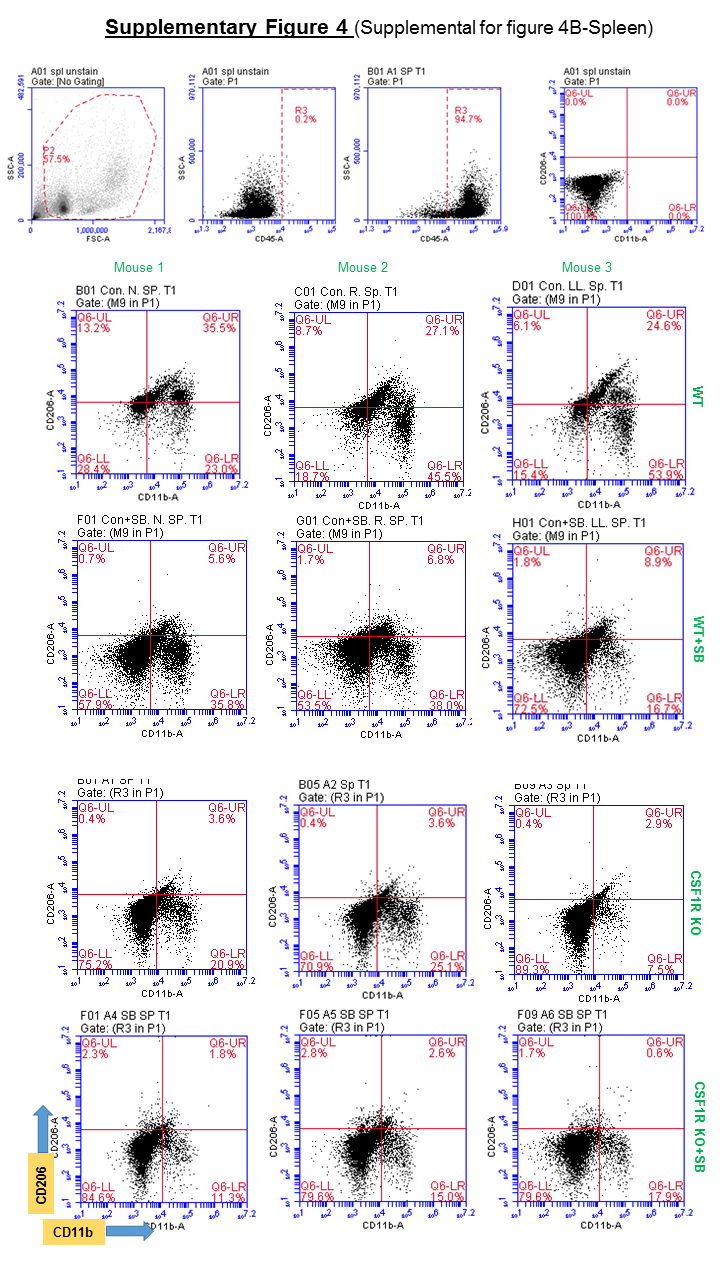
**

**S4 Fig**: (Corresponds Fig 4) Representative dot plots of flow-cytometric analysis showing a significant decrease of TAMs (CD45+CD11b+CD206+) cells in the spleen of the KO mice while a decline was more prominent in the KO mice treated with SB225002.


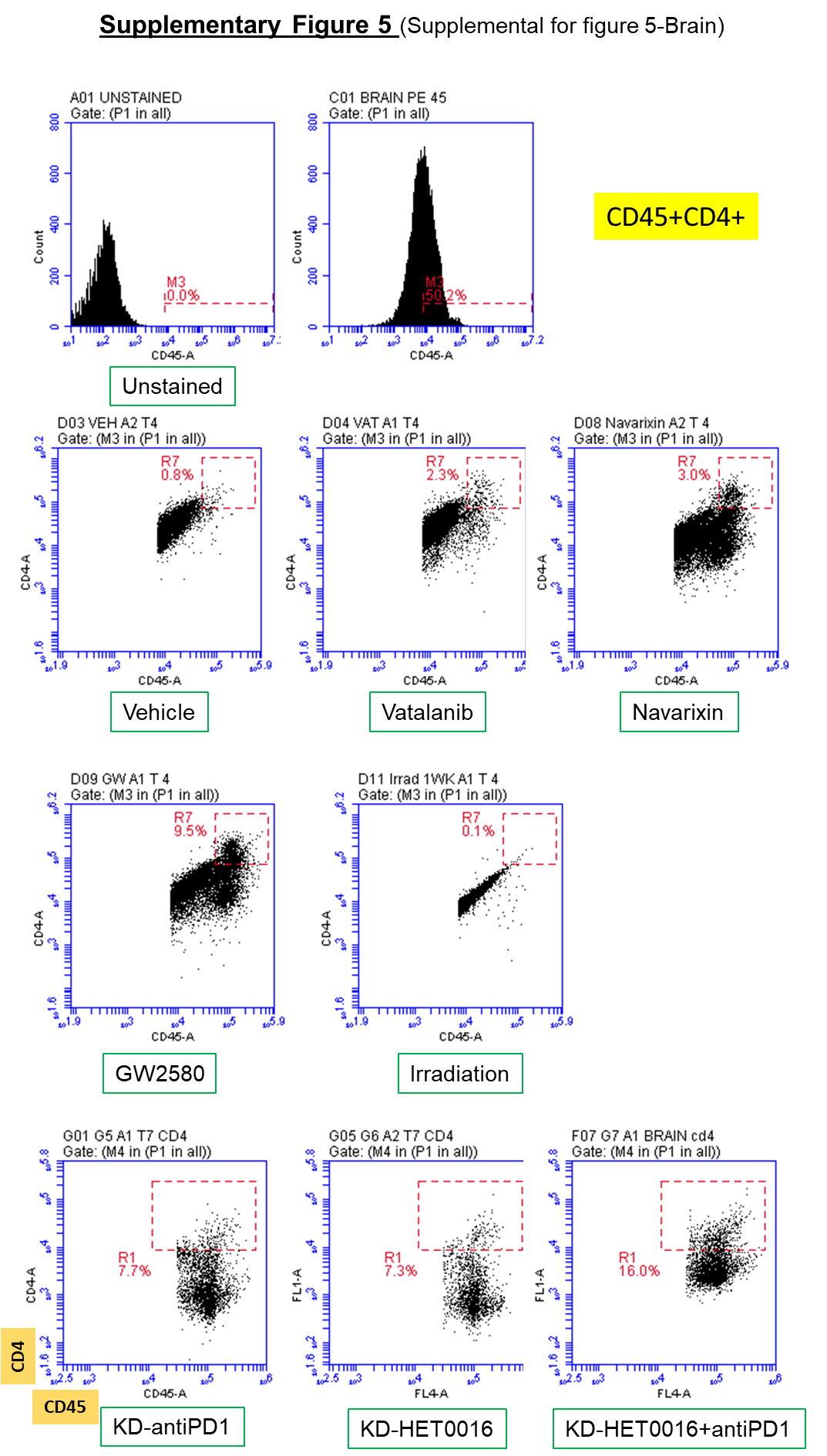


**S5 Fig**: (Corresponds Fig 5) Representative histograms and dot plots of flow-cytometric analysis for CD4+ T-cells in the brain following different treatments in GL261 glioma mouse model.


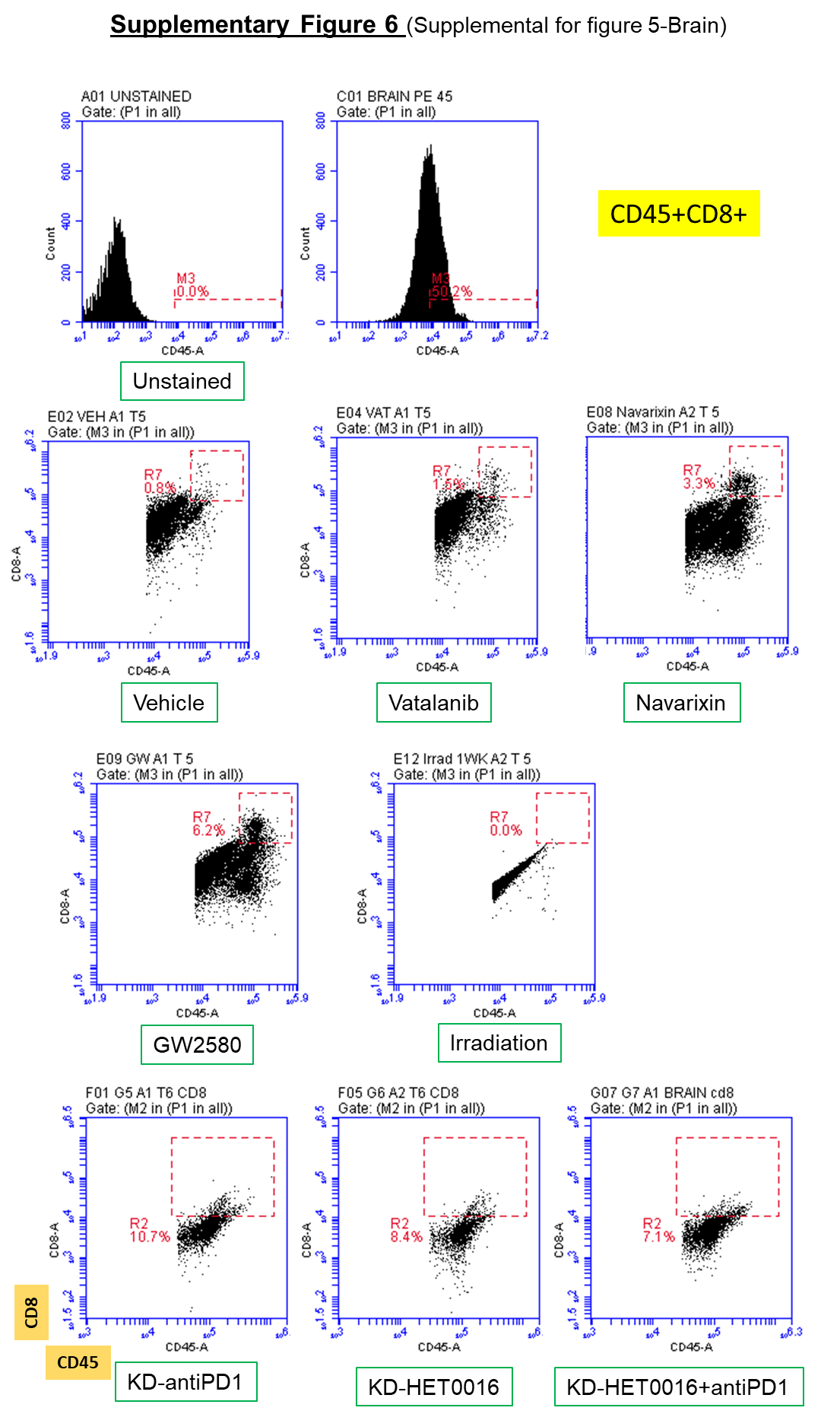


**S6 Fig**: (Corresponds Fig 5) Representative histograms and dot plots of flow-cytometric analysis for CD8+ T-cells in the brain following different treatments in GL261 glioma mouse model.


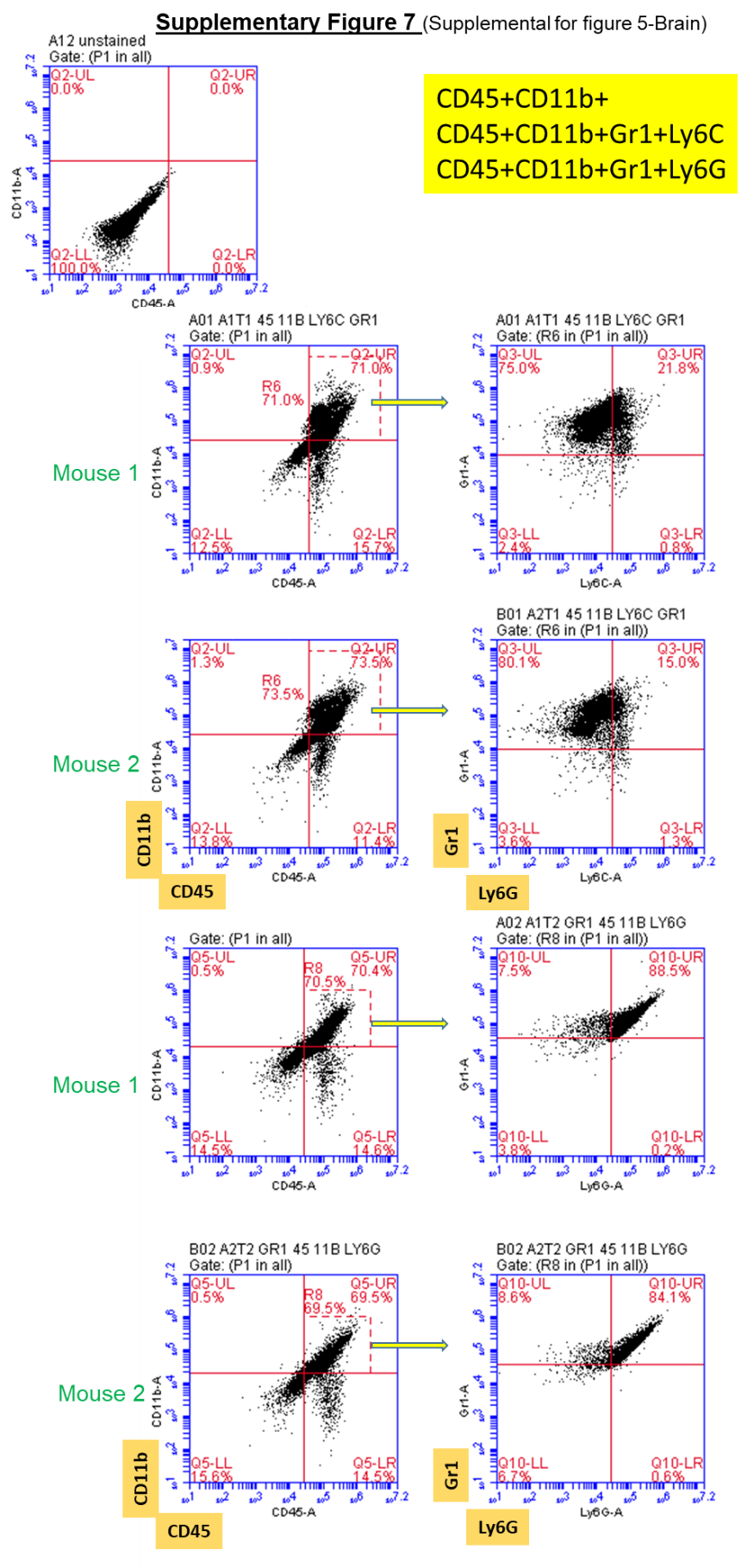


**S7 Fig**: (Corresponds Fig 5) Representative dot plots of gating strategy and flow-cytometric analysis for CD11b+ myeloid cells and MDSCs in the brain following TMZ treatments in GL261 glioma mouse model.

**
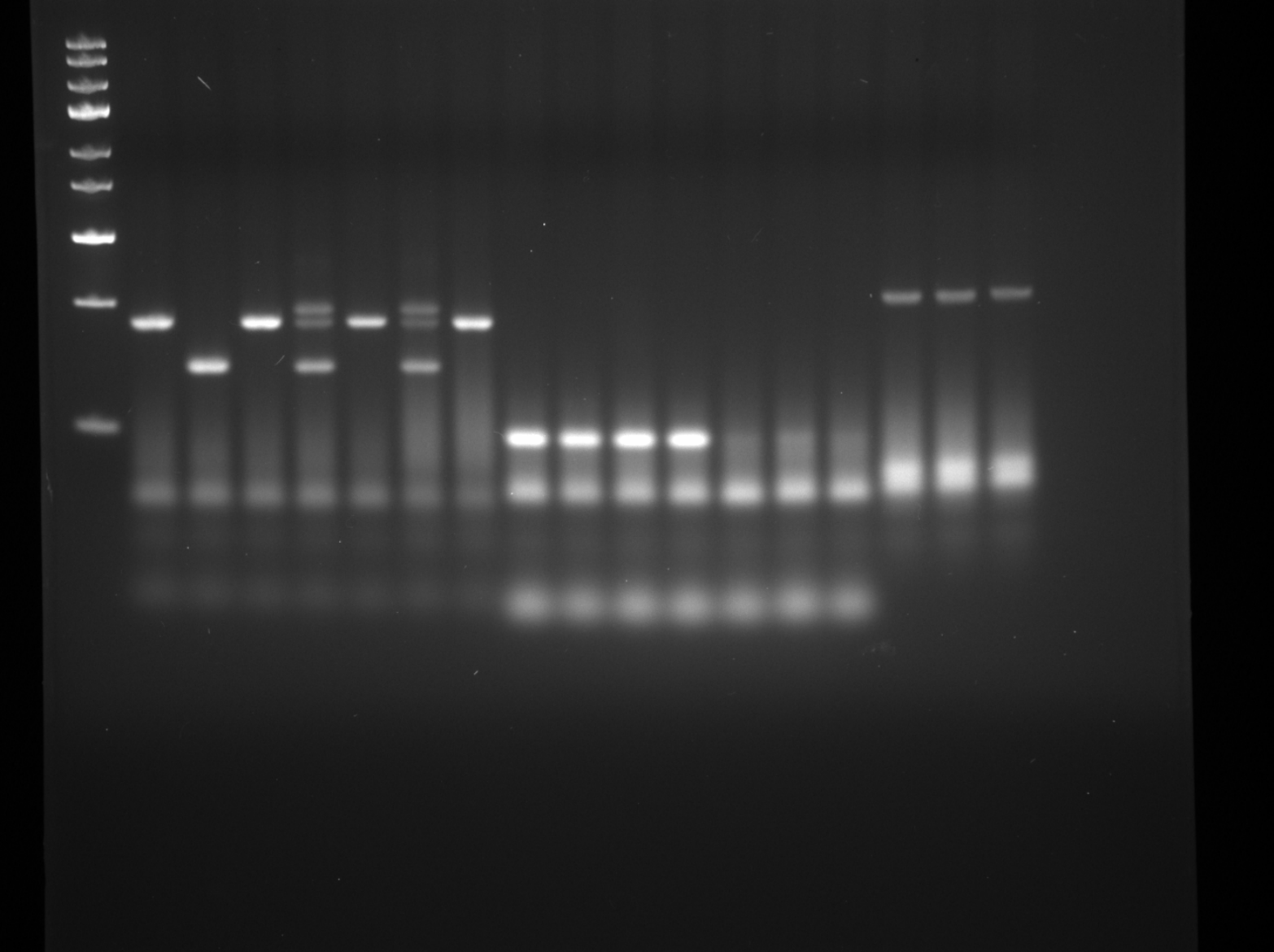
Original agarose blot (full) used for Fig 1A**.

**HET0016 synthesis strategies**

HET0016 and its novel analog were synthesized from commercially available substituted anilines (10 mmol) by treating those with a slight excess of dimethylformamide dimethyl-acetal (11 mmol) in refluxing toluene (20 mL) for 3 hours. After the reaction mixture cooled down, the organic solvent was evaporated in vacuo followed by the treatment of the reaction mixture with the excess of hydroxylamine hydrochloride (30 mmol) in 20 mL of MeOH at room temperature overnight (1,2). The reaction product was then filtered off and purified by recrystallization from EtOH: H_2_O 2:1, filtered and dried under vacuum to give final products as white solids.

1. <https://www.sciencedirect.com/science/article/pii/S0960894X0100614X?via%3Dihub>
2. [Polycyclic N‐hetero compounds. XXXVI. Syntheses and antidepressive evaluation of 11,13,15,17‐tetraazasteroids and their 17‐oxides - Hirota - 1991 - Journal of Heterocyclic Chemistry - Wiley Online Library](https://onlinelibrary.wiley.com/doi/abs/10.1002/jhet.5570280209)

General synthesis scheme for HET0016 and its novel analogs.
